# Surgical Apgar score could predict complications after esophagectomy: a systematic review and meta-analysis

**DOI:** 10.1093/icvts/ivac045

**Published:** 2022-03-16

**Authors:** Chao Zheng, Chao Luo, Kai Xie, Jiang-Shan Li, Hai Zhou, Li-Wen Hu, Gao-Ming Wang, Yi Shen

**Affiliations:** 1 Department of Cardiothoracic Surgery, Jinling Hospital, School of Medicine, Southeast University, Nanjing, China; 2 Department of Cardiothoracic Surgery, Jinling Hospital, Southern Medical University, Guangzhou, China; 3 Department of Cardiothoracic Surgery, Jinling Hospital, Jinling School of Clinical Medicine, Nanjing Medical University, Nanjing, China; 4 University of Science and Technology Beijing, Beijing, China; 5 Department of Cardiothoracic Surgery, Nanjing second Hospital, Medical School of Southeast University, Nanjing, China; 6 Department of Cardiothoracic Surgery, Jinling Hospital, Medical School of Nanjing University, Nanjing, China; 7 Department of Thoracic Surgery, Xuzhou Clinical School of Xuzhou Medical University, Xuzhou, China; 8 Department of Thoracic Surgery, Xuzhou Central Hospital, Xuzhou, China

**Keywords:** surgical Apgar score, esophagectomy, complication, meta-analysis

## Abstract

**OBJECTIVES:**

Esophagectomy is the most effective treatment for oesophageal cancer, although the incidence of postoperative complications remains high. Severe major complications, such as intrathoracic anastomotic leakage, are costly and life-threatening to patients. Therefore, early identification of postoperative complications is essential. The surgical Apgar score (SAS) was introduced by Gawande and colleagues to predict major complications after oesophagectomy. Several studies were carried out with inconsistent results.

**METHODS:**

PubMed, Embase, Web of Science, ClinicalTrials.gov and the Cochrane Library were searched for studies regarding SAS and oesophagectomy. Forest plots were generated using a random-effects model to investigate the actual predictive value of SAS in identifying major complications after oesophagectomy.

**RESULTS:**

Nine retrospective cohort studies were finally identified from selected electronic databases. The meta-analysis demonstrated that SAS could forecast the incidence of postoperative complications (odds ratio = 1.82, 95% confidence interval: 1.43–2.33, *P* < 0.001). Subgroup analysis validated the predictive value of SAS whether as continuous or discrete variables. In addition, a meta-analysis of 4 studies demonstrated that SAS could predict the incidence of pulmonary complications (odds ratio = 2.32, 95% confidence interval: 1.61–3.36, *P* < 0.001). Significant heterogeneity but no publication bias was found.

**CONCLUSIONS:**

Lower SAS scores could predict the incidence of major morbidities and pulmonary complications after oesophagectomy. Significant heterogeneity limits the reliability of the results, even if publication bias is not observed. More high-quality prospective research should be conducted to verify the findings. PROSPERO registration ID: CRD42020209004.

**Figure:**
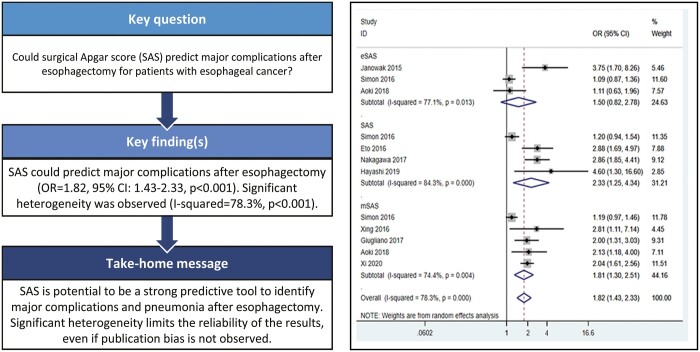

## INTRODUCTION

Oesophageal cancer is one of the most threatening cancers with high morbidity and mortality worldwide[1]. Esophagectomy with two- or three-field lymph node dissections is regarded as an effective surgical therapy for oesophageal cancer. However, postoperative complications are still common due to the highly invasive nature of the procedure[2], despite the development of Enhanced Recovery After Surgery protocols [3], which include perioperative nutritional support, minimally invasive oesophagectomy, improved anaesthesia procedures and individualized analgesia plans [4–6]. Severe complications, such as anastomotic leakage and acute respiratory distress syndrome, always need invasive interventions and might lead to postoperative death. Therefore, early identification of patients at high risk for postoperative complications should be a vital part of the Enhanced Recovery After Surgery programme. In recent decades, several approaches to evaluate the major morbidities after oesophagectomy were introduced, including the Physiologic and Operative Severity Score for the Enumeration of Mortality [7], the Estimation of Physiologic Ability and Surgical Stress [8], and the National Surgical Quality Improvement Programme (NSQIP) [9]. However, these scoring models have not been widely used in practice, because they require complex formulas, and many perioperative parameters are difficult to obtain.

In 2007, Gawande and his colleagues first introduced the concept of the surgical Apgar score (SAS), a 10-point scoring model using 3 variables, estimated blood loss (EBL), lowest mean arterial pressure and lowest heart rate, which are readily acquired during the operation [10]. SAS was then applied in several types of surgical operations, especially those involving digestive cancers [11, 12]. The modified SAS (mSAS), adjusted by EBL, was also found to be significantly associated with postoperative complications in urology and gynecology [13]. Recently, SAS and oesophagectomy SAS (eSAS) were increasingly applied in patients undergoing oesophagectomy. The differences between SAS, mSAS and eSAS are shown in **Table 1**. In previous studies, whether the SAS score could accurately predict the incidence of major complications after oesophagectomy remained controversial. Thus, a systematic review and meta-analysis is urgently required. As far as we know, this study is the first meta-analysis on this topic.

**Table 1:** Different versions of the surgical Apgar score

**A. Surgical Apgar score**					
	0 points	1 point	2 points	3 points	4 points
Estimated blood loss (mL)	>1000	601-1000	101-600	≤100	–
Lowest mean arterial pressure (mm Hg)	<40	40-54	55-69	≥70	–
Lowest heart rate (beats/min)	>85	76-85	66-75	56-65	≤55
**B. The modified surgical Apgar score**					
	0 points	1 point	2 points	3 points	4 points
Estimated blood loss (mL)	>75th percentile	>Median-75th percentile	>25th percentile-Median	≤25th percentile	–
Lowest mean arterial pressure (mmHg)	<40	40-54	55-69	≥70	–
Lowest heart rate (beats/min)	>85	76-85	66-75	56-65	≤55
**C. The oesophagectomy surgical Apgar score**					
	0 points	1 point	2 points	3 points	4 points
Estimated blood loss (mL)	>300	201-300	151-200	≤150	–
Lowest mean arterial pressure (mm Hg)	<40	40-54	55-69	≥70	–
Lowest heart rate (beats/min)	>85	76-85	66-75	56-65	≤55

Surgical Apgar score = sum of the points for the 3 variables during an oesophagectomy.

## MATERIALS AND METHODS

### Overview

This systematic review and meta-analysis was registered on PROSPERO (https://www.crd.york.ac.uk/PROSPERO/, the registration ID: CRD42020209004), with a prespecified analysis plan.

### Search strategy

A systematic literature search was conducted in the following databases: PubMed, Embase, Web of Science, ClinicalTrials.gov and the Cochrane Library as of 15 December 2020. Only articles published in English were eligible. Observational cohort studies and clinical trials would be taken into consideration. Medical subject headings (MeSH) were used in PubMed and Embase, respectively. The search strategies and terms in PubMed were as follows: (Esophagectomy [MeSH] OR Esophagus [MeSH] OR oesophagus [Title/Abstract] OR oesophagus [Title/Abstract] OR oesophageal [Title/Abstract] OR oesophageal [Title/Abstract] OR oesophagectomy [Title/Abstract] OR oesophagectomy [Title/Abstract]) AND (“Apgar score” [MeSH] OR “surgical Apgar score” [All Fields] OR SAS [All Fields] OR “modified surgical Apgar score” [All Fields] OR mSAS [All Fields] OR “oesophagectomy surgical Apgar score” [All Fields] OR eSAS [All Fields]).

### Eligibility criteria

The eligible criteria were as follows: (i) observational studies or randomized controlled trials; (ii) studies containing short-term outcomes with sufficient data; (iii) the latest research that was based on the same cohort; and (iv) papers published in English. Studies were excluded when article type was case report, review, abstract, animal experiment or conference report.

### Data extraction

The process of data extraction was carried out independently by 2 reviewers (Kai Xie, Jiang-Shan Li). The extracted data were as follows: first author, publication year, country, number of patients, the definition of morbidity, SAS score, clinical outcomes, odds ratio (OR) with 95% confidence interval (CI). Primary outcome was defined as major morbidity after oesophagectomy, and secondary outcomes included pulmonary complications, perioperative mortality and 5-year survival rate.

### Definition of major comorbidity

Major morbidity was defined according to the standard of the Clavien-Dindo classification criteria [14], and/or the NSQIP, and/or Esophagectomy Complications Consensus Group guidelines [15], and/or thoracic morbidity and mortality classification system (based on the Clavien-Dindo classification criteria) [16] in the 9 cohort studies. Of these, the Clavien-Dindo classification criteria (grade III or higher) were applied most frequently.

### Quality assessment

The Jadad scale and the Newcastle-Ottawa Scale (NOS) were utilized to assess the quality of randomized controlled trials and cohort studies, respectively [17]. The risk of bias assessment was conducted by 2 reviewers independently (Chao Luo, Kai Xie), and any disagreements were settled by discussion and the intervention of a third reviewer (Chao Zheng).

### Statistical analysis

STATA 16.0 software (Stata Corp, College Station, TX, USA) was applied in data analyses according to the Preferred Reporting Items for Systematic Reviews and Meta-Analyses guidelines [18]. Forest plots based on a random-effects model were generated to characterize the predictive efficacy of SAS for postoperative complications. Pooled ORs with 95% CIs were adopted for the comparison. Heterogeneity was evaluated using χ^2^-based Q statistics and the I^2^ test. Sensitivity analyses using sequential removal of 1 study, subgroup analyses and meta-regression were conducted to explore the source of heterogeneity if significant heterogeneity (I^2^≥50% or *P* < 0.05) was found. A funnel plot was generated to assess publication bias, and Egger’s test and Begg’s test [19] were carried out if necessary. A *P*-value of 0.05 was set as the significant threshold.

## RESULT

### Literature search and characteristics

**Figure 1:**
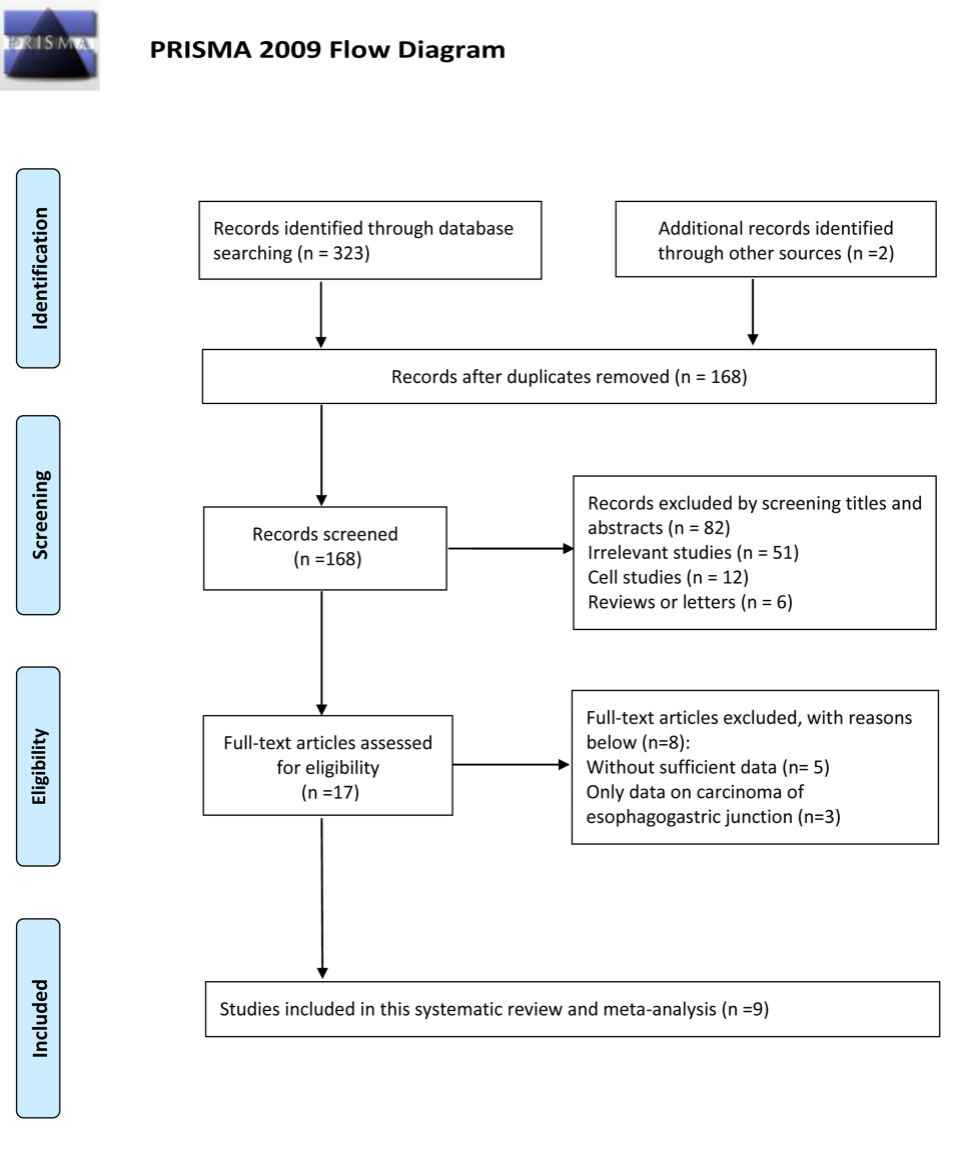
Flow diagram of the literature retrieval according to Preferred Reporting Items for Systematic Reviews and Meta-Analyses guidelines.

According to the Preferred Reporting Items for Systematic Reviews and Meta-Analyses guidelines, the selection flow diagram is shown in **Fig. 1**. After systematic retrieval of literature from the selected electronic databases, a total of 325 citations were searched. Nine cohort studies [20–28] were eventually included in this review after eliminating duplicates and screening for full-text articles, with the basic characteristics presented in **Table 2**. Overall, a total of 2,232 patients were included in the review; major postoperative complications occurred in 904 of these patients. Six of 9 cohort studies reported the SAS as a discrete variable whereas the remaining 3 studies reported it as a continuous variable. Except for major morbidity, 4 studies reported the predictive value of SAS in predicting pneumonia or pulmonary complications after oesophagectomy. In addition, Giugliano *et al.* [24] reported the association between SAS and perioperative mortality (OR = 0.40, 95% CI: 0.19–0.86, *P* = 0.019)., Nakagawa *et al.*[27] reported that, for patients with clinical stages II, III and IV oesophageal cancers, a lower SAS score (≤5 vs >5) was associated with a lower 5-year survival rate (HR = 1.57, 95% CI: 1.05–2.35, *P* = 0.029).

**Table 2:** Basic characteristics of included studies

Study	N	Country	Version of SAS	Definition of morbidity	Comparison	OR (95% CI)	
Janowak 2015 [26]	168	United States	eSAS	NSQIP+C-D	≤6 vs >6	3.75 (1.70-8.26)	
Strøyer 2016 [20]	234	Denmark	SAS	C-D	Continuous	0.83 (0.65-1.07)	
			mSAS		Continuous	0.84 (0.68-1.03)	
			eSAS		Continuous	0.92 (0.74-1.15)	
Eto 2016 [23]	399	Japan	SAS	C-D	≤5 vs >5	2.88 (1.68-4.97)	
Xing 2016 [28]	189	China	mSAS	TM&M	≤7 vs >7	2.81 (1.11-7.14)	
Giugliano2017 [24]	212	United States	mSAS	C-D	Continuous	0.50 (0.33-0.76)	
Nakagawa2017 [27]	400	Japan	SAS	C-D	≤5 vs >5	2.86 (1.85-4.41)	
Aoki 2018 [22]	246	Japan	mSAS	NSQIP+C-D	≤3 vs >3	2.13 (1.18-4.00)	
			eSAS		≤3 vs >3	1.11 (0.63-1.96)	
Hayashi 2019 [25]	190	Japan	SAS	C-D	≤5 vs >5	4.67 (1.30-16.67)	
Xi 2020 [21]	194	China	mSAS	ECCG+C-D	Continuous	0.49 (0.39-0.62)	

SAS: surgical Apgar score; eSAS: oesophagectomy SAS; mSAS: modified SAS; OR: odds ratio; CI: confidence interval; NOS: Newcastle-Ottawa Scale; NSQIP: National Surgical Quality Improvement Programme; C-D: Clavien-Dindo classification criteria; TM&M: thoracic morbidity and mortality classification system; ECCG: Esophagectomy Complications Consensus Group.

### Quality assessment and risk of bias

The risk of bias in each study was evaluated using the NOS scale, presented in **Table 3**. Based on the NOS criteria, 0–3, 4–6 and 7–9 were defined as low, moderate and high quality, respectively. Therefore, 8 cohort studies were classified as high quality; only the study of Strøyer *et al. [20]* was classified as moderate quality.

**Table 3:** The Newcastle-Ottawa scale for individual studies.

Study	Selection				Comparability	Exposure			Total quality scores
		Representativeness of the exposed cohort	Selection of the non-exposed cohort	Ascertainment of exposure	Demonstration that outcome of interest was not present at the start of the study	Comparability of cohorts on the basis of the design or analysis	Assessment of outcome	Was follow-up long enough for outcomes to occur	Adequacy of follow-up of cohorts
Janowak 2015 [26]	★		★	★	★	★	★	★	7
Strøyer 2016 [20]		★		★	★	★	★	★	6
Eto 2016 [23]	★	★		★	★	★	★	★	7
Xing 2016 [28]	★	★	★	★	★	★	★		7
Giugliano2017 [24]	★	★		★	★	★	★	★	7
Nakagawa2017 [27]	★	★	★	★	★	★	★	★	8
Aoki 2018 [22]	★	★	★	★	★	★	★		7
Hayashi 2019 [25]	★		★	★	★	★	★	★	7
Xi 2020 [21]	★	★		★	★	★	★	★	7

Note: ★ represents 1 point.

### Meta-analysis of primary outcome

**Figure 2:**
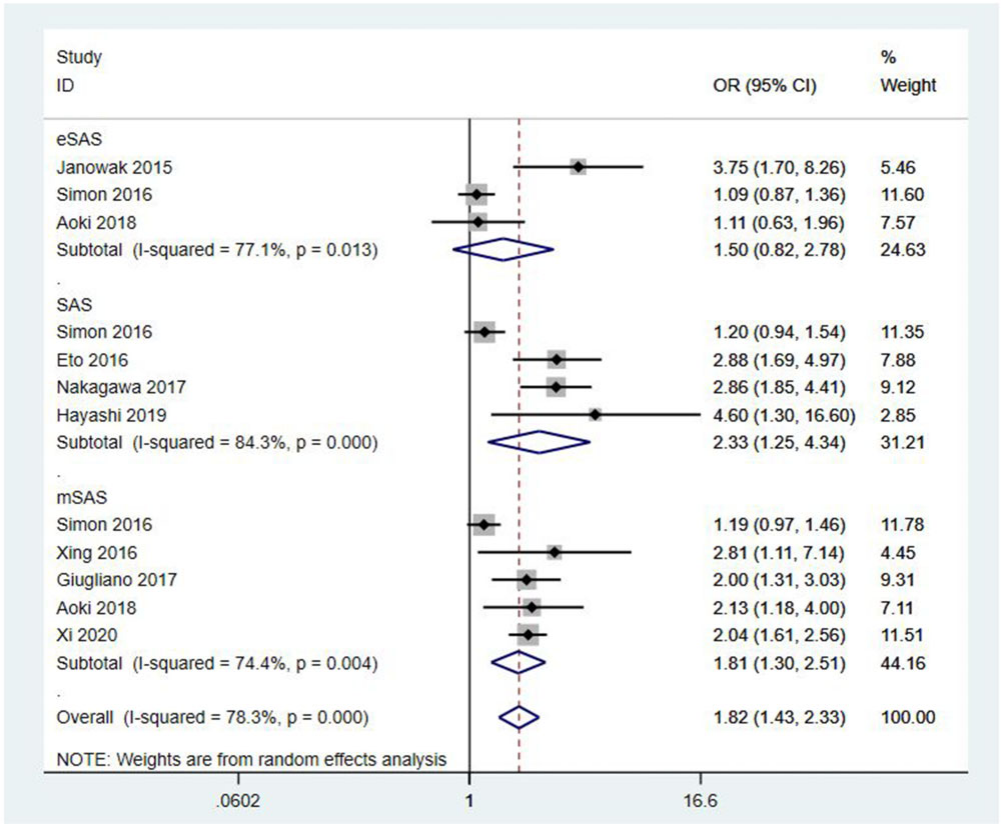
Forest plot of the 9 studies grouped by versions of the surgical Apgar score. OR: odds ratio; CI: confidence interval; SAS: surgical Apgar score; eSAS: oesophagectomy SAS; mSAS: modified SAS.

Due to inconsistencies in the methods of assessing EBL, more than 1 version of the SAS score was introduced in the studies of Strøyer *et al.*[20] and Aoki *et al.*[22]. Thus, the forest plot of the primary outcome was generated and was grouped by the different versions of the SAS score (**Fig. 2**). It demonstrated that SAS could predict major complications after oesophagectomy (OR = 1.82, 95% CI: 1.43–2.33, *P* < 0.001). Significant heterogeneity was observed (I^2^ = 78.3%, *P* < 0.001).

### Sensitivity and subgroup analyses

**Figure 3:**
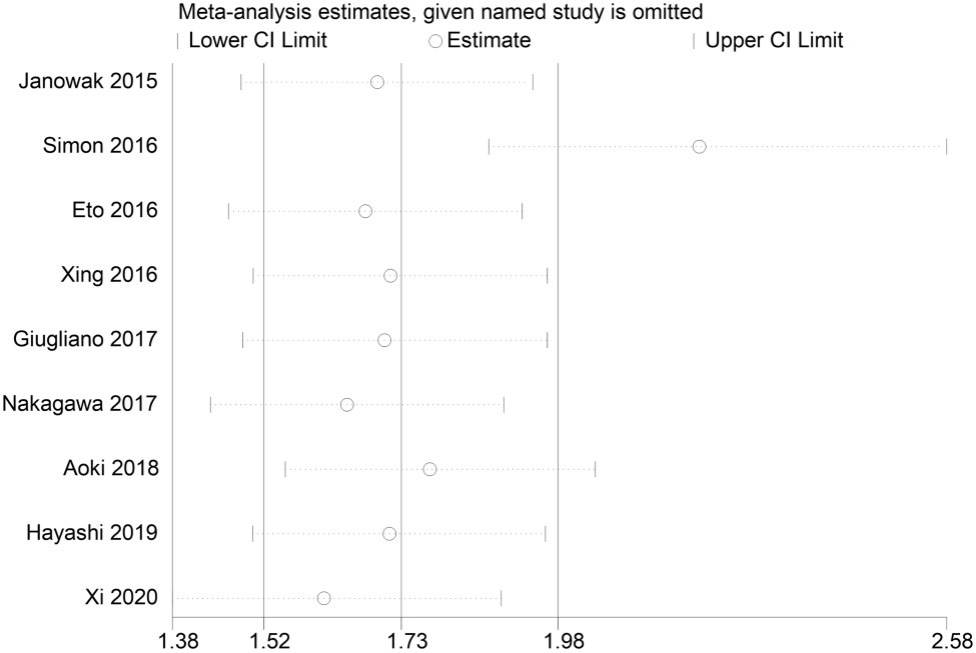
Sensitivity analysis of the 9 included studies. CI: confidence interval.

To investigate the source of heterogeneity, sensitivity analyses were carried out by removing 1 study at a time. The result showed that the study of Strøyer *et al.* might have contributed to the major source of heterogeneity (**Fig. 3**).

**Figure 4:**
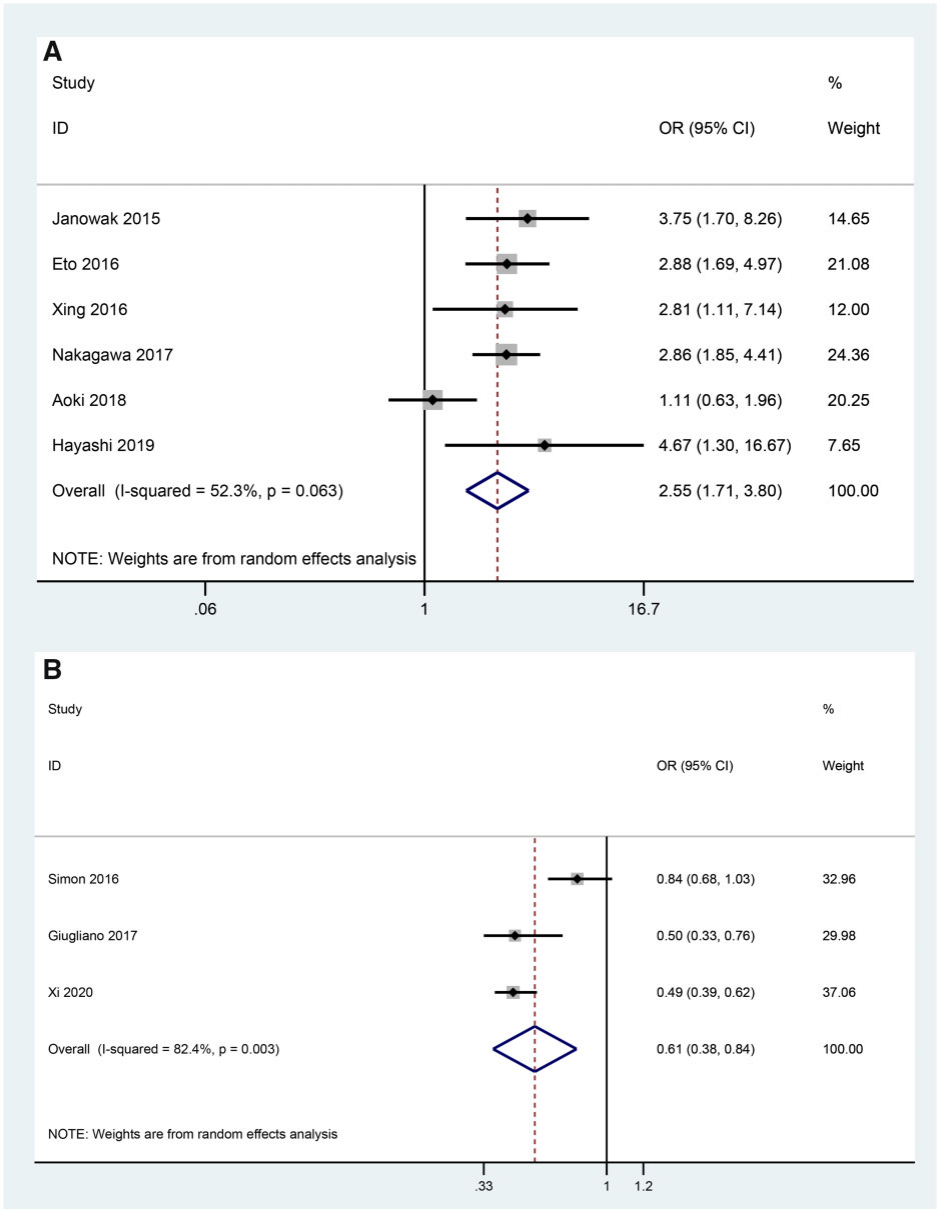
Subgroup analysis grouped by discrete variables (**A**) and continuous variables (**B**). OR: odds ratio; CI: confidence interval.

Considering that 3 studies reported SAS as a continuous variable while the remaining studies reported it as a discrete variable, we further conducted a subgroup analysis. The estimates indicated that patients with higher SAS scores had a higher incidence of major morbidities after oesophagectomy when SAS was pooled as a discrete variable (OR = 2.55, 95% CI: 1.71–3.80, *P* < 0.001) (Fig. 4A). In addition, the probability of postoperative complications decreased by 39% when the patient's SAS score increased by 1 point (OR = 0.61, 95% CI: 0.38–0.84, *P* < 0.001) (**Fig.** 4B).

### Meta-regression

Further meta-regression was performed to find heterogeneity between studies. Mean age, publication year, countries, versions of SAS and numbers of patients were included in the regression model (tau^2^=0, adjusted R^2^=100.00%, *P* = 0.369). The results indicated that none of the 5 covariates was the main source of heterogeneity (Supplementary Fig. 1, all *P* > 0.05).

### Meta-analysis of pulmonary complications

**Figure 5:**
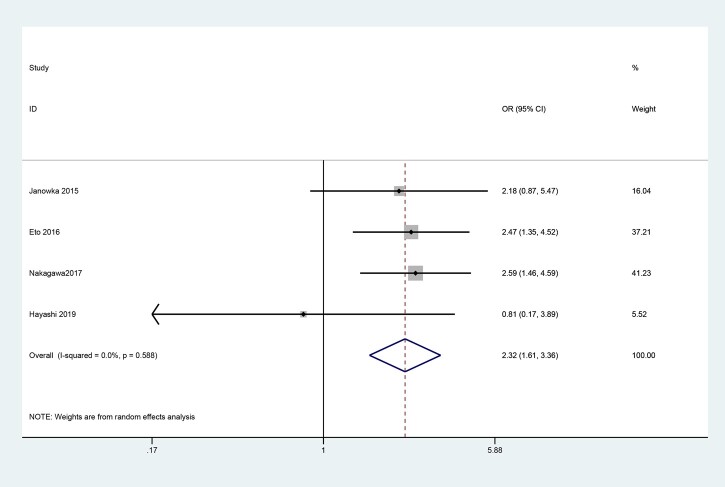
Forest plots of surgical Apgar scores predicting pulmonary complications. OR: odds ratio; CI: confidence interval.

Four cohort studies reported the predictive value of SAS in predicting pneumonia or pulmonary complications after oesophagectomy according to the NSQIP and/or Clavien-Dindo (C-D) criteria, while the results were inconsistent. The forest plot demonstrated that SAS could predict the incidence of pulmonary complications (OR = 2.32, 95% CI: 1.61–3.36, *P* < 0.001) (**Fig. 5**). Heterogeneity was not observed in the forest plot (I-squared = 0.0%, *P* = 0.588).

### Publication bias

**Figure 6:**
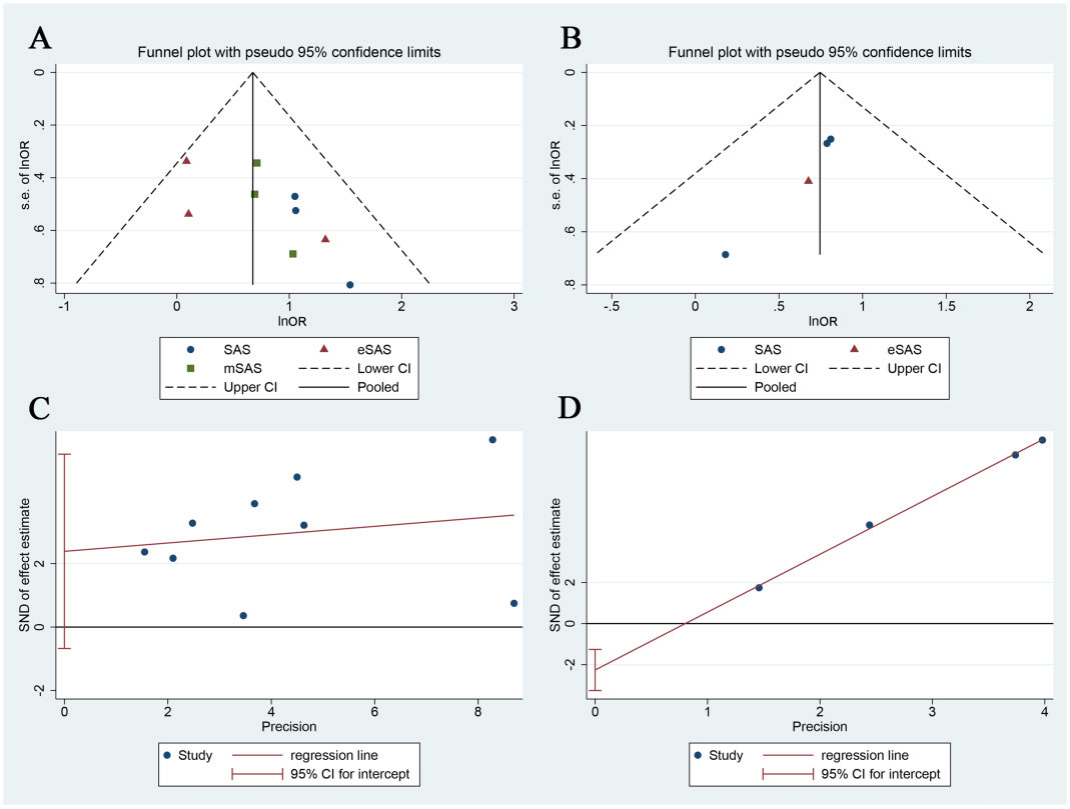
Funnel plot (**A**) and plot of Egger’s test (**B**). s.e. InOR: standard error of lnOR; SAS: surgical Apgar score; mSAS: modified SAS; eSAS: oesophagectomy SAS; CI: confidence interval; SND: standard normal deviate

We carried out funnel plots to investigate the publication bias of primary outcome and pulmonary complications (Fig. 6A–D). Potential publication bias was observed from visual inspection of the asymmetry of the funnel plots for the primary outcome (**Fig.** 6A) and pulmonary complications (**Fig.** 6B). Thus, Egger’s test (**Fig.** 6C) and Begg’s test were applied. The *P*-values (*P* = 0.325, *P* = 0.386, respectively) indicated that no significant publication bias was found for primary outcome. However, significant publication bias of pulmonary complications was observed using Egger’s test (**Fig. 6D**, *P* = 0.010) and Begg’s test (*P* = 0.042).

## DISCUSSION

Several models and scoring systems to predict the incidence of postoperative complications after oesophagectomy were introduced in previous studies. Compared with other complicated models, SAS as reported by Gawande *et al.* comprised 3 intraoperative parameters that are easily accessible. Several studies on the topic of whether SAS could predict major complications were carried out in recent years with inconsistent results. In this meta-analysis, we found that SAS could forecast postoperative complications after oesophagectomy.

Nine cohort studies were included. With different versions of SAS in the included studies, we generated the forest plot classified by the versions of SAS. The result demonstrated that SAS could be a predictive tool in predicting major morbidity for patients who underwent oesophagectomy (OR = 1.82, 95% CI: 1.43–2.33, *P* ≤ 0.001), although significant heterogeneity existed between the studies (I^2^ = 78.3%). Thus, we conducted sensitivity analyses, subgroup analyses and meta-regression to explore the source of heterogeneity. Sensitivity analyses showed that the study of Strøyer *et al.* might contribute to the major source of heterogeneity. Subgroup analyses demonstrated that SAS was an effective predictive tool for major complications after oesophagectomy and that variable types might be one of the sources of heterogeneity. Furthermore, we conducted a meta-regression model in which mean age, publication year, countries, versions of SAS and numbers of patients were included: none of them contributed to the major source of heterogeneity.

Considering that pulmonary infection was associated with long-term prognosis in patients with oesophageal cancer [29, 30], we investigated whether SAS could predict pneumonia or pulmonary complications. A meta-analysis of 4 studies indicated that SAS could predict pneumonia or pulmonary complications (OR = 2.33, 95% CI: 1.61–3.37, *P* < 0.001). Identification of patients at high risk stratified by SAS might be helpful in improving patients’ long-term outcomes. In addition, Kofoed and his colleagues [31] reported that anastomotic leakage was a prognostic factor for long-term survival whereas the association between SAS and anastomotic leakage was reported in only 2 studies, with inconsistent results. Moreover, the association between SAS and other clinical outcomes, including perioperative mortality and the 5-year survival rate, was reported in only 1 study. More high-quality studies conducted in high-volume institutions are needed to verify the value of SAS and make it more applicable to clinical practice.

Significant heterogeneity was observed in the meta-analysis, which might be the result of several factors. Primarily, different versions of SAS were applied considering different amounts of estimated blood loss. In the study of Strøyer *et al.*, 3 versions of SAS (SAS, eSAS, mSAS) were applied, and none of them showed predictive value in predicting major morbidity after oesophagectomy. In addition, the definition of major morbidity was not consistent in the 9 studies, which might cause selection bias. Moreover, some studies reported Ivor-Lewis and/or McKeown oesophagectomy, whereas some studies reported open oesophagectomy, minimally invasive oesophagectomy and hybrid oesophagectomy. Differences in approaches of oesophagectomy might result in heterogeneity between studies. Last but not least, although most of the included studies reported SAS as a discrete variable, the cut-off values of SAS in the studies were different because there is no internationally accepted cut-off value.

The SAS score contains only intraoperative variables, including EBL, lowest mean arterial pressure and lowest heart rate, which are associated with adverse short-term outcomes [32, 33]. Traction of the mesentery during oesophagectomy would lead to hypotension, tachycardia and flushing, which is also called mesenteric traction syndrome [34]. Mesenteric traction syndrome may be an important reason why SAS can be used to predict postoperative complications. However, a variety of other factors could affect the incidences of postoperative complications. Nakashima *et al.* [35] reported that sarcopenia was significantly associated with anastomotic leakage for elderly patients with oesophageal cancer. Our team previously found that the ratio of gastric tube length to thorax length was a vital factor in anastomotic leakage [36]. A recent meta-analysis reported that preoperative nutritional support was beneficial to reduce the incidence of infectious complications [37]. Therefore, prediction of complications after oesophagectomy is a complex procedure, and many factors should be considered.

The advantages of this meta-analysis are as follows: Although several related studies were done since the introduction of SAS, whether SAS could predict major complications remained unclear because of inconsistent results. As far as we know, this is the first systematic review and meta-analysis on this theme. In addition, we found that the SAS score could predict the incidence of pneumonia or pulmonary complications. Only retrospective cohort studies were included in the meta-analysis with the ineluctable limitation of selection bias, which is one of the limitations of this research. Besides, though SAS comprised 3 intraoperative variables, it was not widely applied in clinical practice because many researchers and thoracic surgeons believed that intraoperative parameters could only reflect the short-term status of patients. Only 3 studies have reported SAS as a continuous variable, and more studies are needed to verify the linear relationship between SAS and complications.

## CONCLUSION

SAS has the potential to be a strong predictive tool to identify major complications and pulmonary complication after oesophagectomy in the early phase, decreasing complication-related costs and postoperative deaths. Significant heterogeneity limits the reliability of the results, even if publication bias is not observed. More high-quality prospective studies are needed to verify and update the findings.

**Conflict of Interest**: None declared.

## DATA Availability

The authors declare that the data supporting the findings are collected from the 9 included studies and can be found in the article.

## FUNDING

This study is supported by the National Natural Science Foundation of China (No. 81702444), the Medical Scientific Research Project of Jiangsu Health Commission (No. ZD2021011) and the Excellent Talents Fund Project of Xuzhou Medical University (No. XYFY2020017).

## Supplementary Material

ivac045_Supplementary_Data
