# Supplementary figures and images for: Surgical Apgar score could predict complications after esophagectomy: a systematic review and meta-analysis
Source: Interact Cardiovasc Thorac Surg. 2022 Mar 16;35(1):ivac045. doi: 10.1093/icvts/ivac045 (PMC9714643; doi:10.1093/icvts/ivac045)

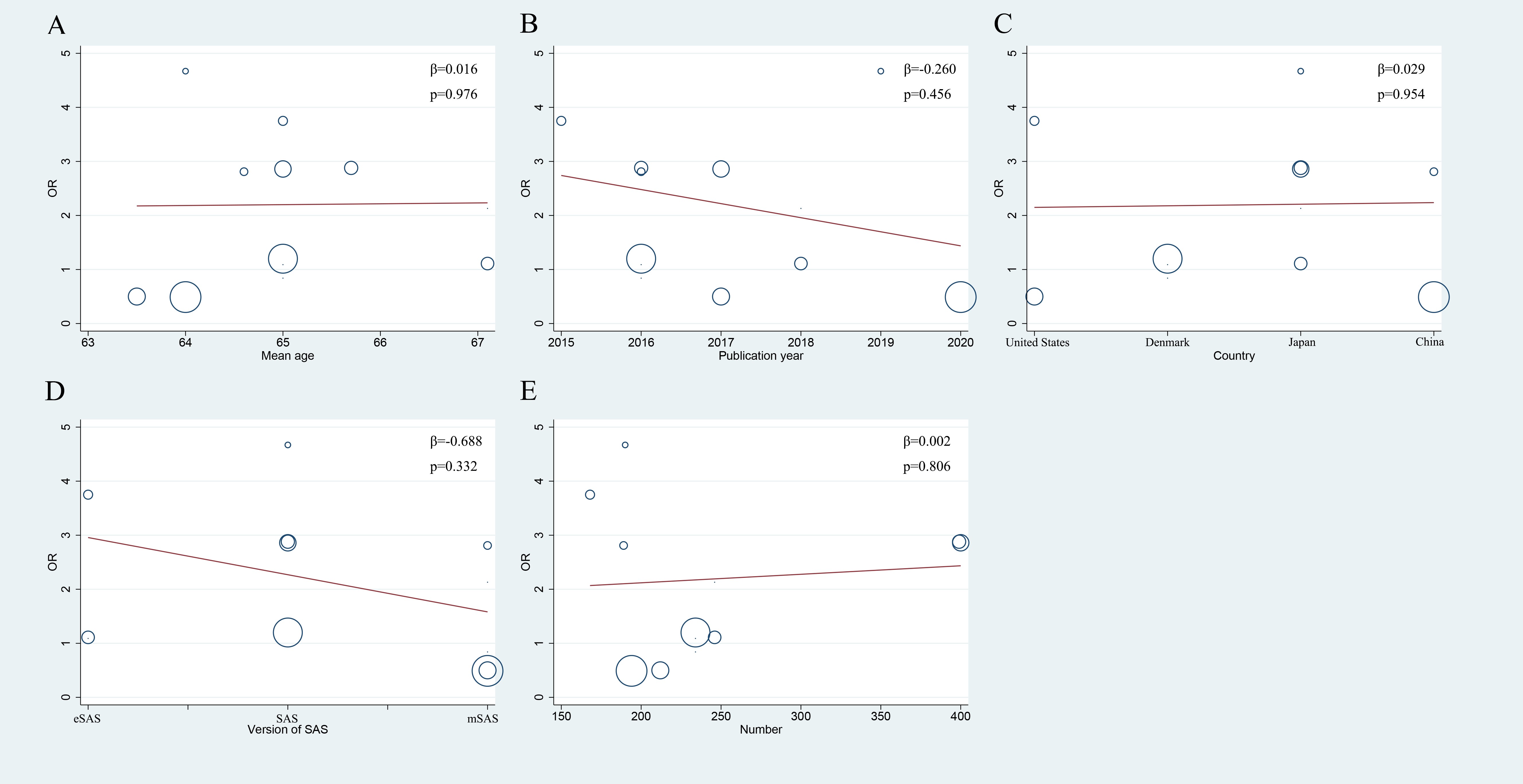

Supplement: ivac045_Supplementary_Data [file ivac045_supplementary_data.jpeg]
